# Antimicrobial use practices in canine and feline dental procedures performed in primary care veterinary practices in the United States

**DOI:** 10.1371/journal.pone.0295070

**Published:** 2023-12-08

**Authors:** J. Scott Weese, Ian Battersby, JoAnn Morrison, Nathaniel Spofford, Maria Soltero-Rivera

**Affiliations:** 1 Department of Pathobiology, Ontario Veterinary College, University of Guelph, Guelph, ON, Canada; 2 Mars Veterinary Health, Vancouver, WA, United States of America; 3 Banfield Pet Hospital, Vancouver, WA, United States of America; 4 Department of Veterinary Surgical and Radiological Sciences, University of California, Davis, CA, United States of America; University of Puthisastra, CAMBODIA

## Abstract

This study examined the utilization of antimicrobials in canines and felines receiving dental treatments in veterinary clinics in the United States, retrospectively. A total of 818,150 animals (713,901 procedures in dogs and 104,249 procedures in cats) underwent dental procedures under general anesthesia in 2020. These included dental prophylaxis and extractions. Patient demographic data, antimicrobial treatment, treatment duration, dose, periodontal disease score, whether tooth extractions were performed and how many extractions were performed was recorded. Our results showed that local or systemic antimicrobials were used in 116,723/713,901 (16.4%) procedures in dogs and 14,264/104,249 (14%) procedures in cats. Age, weight, extraction of one or more teeth and diagnosis of periodontal disease (any stage) were associated with increased likelihood of antimicrobial administration using univariable analysis (all P<0.001) and in the multivariable model. Clindamycin, amoxicillin-clavulanate and amoxicillin were the most common oral antimicrobials used in dogs and cats. Drugs classified as highest priority clinically important antibiotics (HPCIA) were administered to 30,960/116,723 (26.5%) of dogs and 7,469/14,264 (52%) of treated cats. The results obtained can inform interventions to optimize patient care and promote prudent use of antimicrobials during dental procedures in canine and feline patients.

## Introduction

As the silent pandemic of antimicrobial resistance continues to expand and the impacts on human and animal populations become clearer, efforts to optimize antimicrobial use (AMU) are increasing. Antimicrobial stewardship involved myriad approaches to address AMU, with a core component being surveillance and understanding the reasons why antimicrobials are being prescribed. A clear understanding of antimicrobial use practices is required to evaluate current practices, to identify areas for improvement, to identify knowledge gaps and to develop interventions [1,2].

Dental disease is common in dogs and cats [3,4], and dental procedures are amongst the most common procedures performed in veterinary clinics. Antimicrobials may be used to prevent infection at distant sites due to bacteremia [5], to reduce local infections associated with surgery [6], or to treat oral infections [7–10]. Anecdotally, antimicrobials are often used prophylactically during dental procedures but this has been minimally investigated. Given the volume of procedures that are performed, veterinary dentistry is an important area to include in antimicrobial stewardship efforts because of the potential for use of large volumes of antimicrobials. The objective of this study was to analyse antimicrobial use practices in dogs and cats undergoing dental procedures at a network of veterinary clinics in the United States.

## Materials and methods

An electronic medical record search was performed to identify dogs and cats that underwent dental procedures Banfield Pet Hospitals, the largest primary care veterinary practice in the United States during January to December of 2020. All hospitals utilize the same proprietary medical record system (PetWare®). Structured and unstructured data are downloaded nightly from all hospitals to a centralized data warehouse, making large amounts of data available for analysis. Dental procedures met inclusion criteria when specific inventory line items denoting procedures such as dental prophylaxis and extractions were identified. All dental prophylaxes would have been done with general anesthesia, which is part of the procedure package and cost. Invoiced extraction codes were used to determine whether, which, and how many teeth were extracted. Structured pharmaceutical data include drug name, concentration, route of administration, dose, and duration of treatment. Animal species, age, weight, antimicrobial treatment, treatment duration, dose, periodontal disease severity as per AVDC staging (PD0 –no disease present, PD1- gingivitis with no attachment loss, PD2 - <25% attachment loss, PD3–26–50% attachment loss, PD4 - >51% attachment loss) [11], whether tooth extractions were performed and how many extractions were performed was obtained. Cases missing any of the data points mentioned above were excluded from this study. Presence of other dental diseases such as those leading to loss of tooth substance (i.e., tooth resorption, carious lesions) or endodontal disease (i.e., intrinsically discolored, or fractured teeth) were not recorded in this study. Written consent was obtained from clients for every pet included in the analysis, prior to treatment. Institutional Review Board approval was not required for this study as there is no access to client data and this study qualifies as quality assurance and quality improvement activities.

Antimicrobials and antimicrobial combinations that accounted for <1% in both dogs and cats were classified as ‘other’ for some analyses. Antimicrobials were classified according to the World Health Organization’s Critically Important Antimicrobial categorization.^3^

Descriptive statistics were used to describe the data. Distributions were visually examined. Univariable analysis was performed using chi-squared tests (categorical data) and linear regression (continuous data). Variables with a *P*<0.20 were included in the multivariable model. Stepwise backwards logistic regression was performed, retaining variables with *P*<0.05 in the final model. Odds ratios and 95% confidence intervals were calculated. Fit of the final model was assessed. Analysis was performed using JMP 16.2 (SAS Institute Inc, Cary, NC, USA).

Duration of treatment was described for treatments that were continued after the procedure. Because of the common use of the highly protein bound drug cefovecin, which provides prolonged drug levels and lack of a standard approach for inclusion of it in analysis of duration, duration analysis was descriptive and only included oral antimicrobials. The number of procedures performed per clinic was categorized and Steel-Dwass test was used to evaluate the association of clinic size and antimicrobial administration.

## Results

A total of 818,150 dental procedures were performed in 2020 at 1,076 veterinary clinics in 42 US states, plus the District of Columbia and Puerto Rico. This consisted of 713,901 procedures being performed in dogs and 104,249 procedures being performed in cats. The median age of dogs was 7.5y (interquartile range (IQR), 5y). Median weight of dogs was 9.3 kg (IQR 19 kg). The median age of cats was 7.6y (IQR 5.9y). Cat weight was a median of 5.2 kg (IQR 1.8kg).

### Canine patients

**Fig 1:**
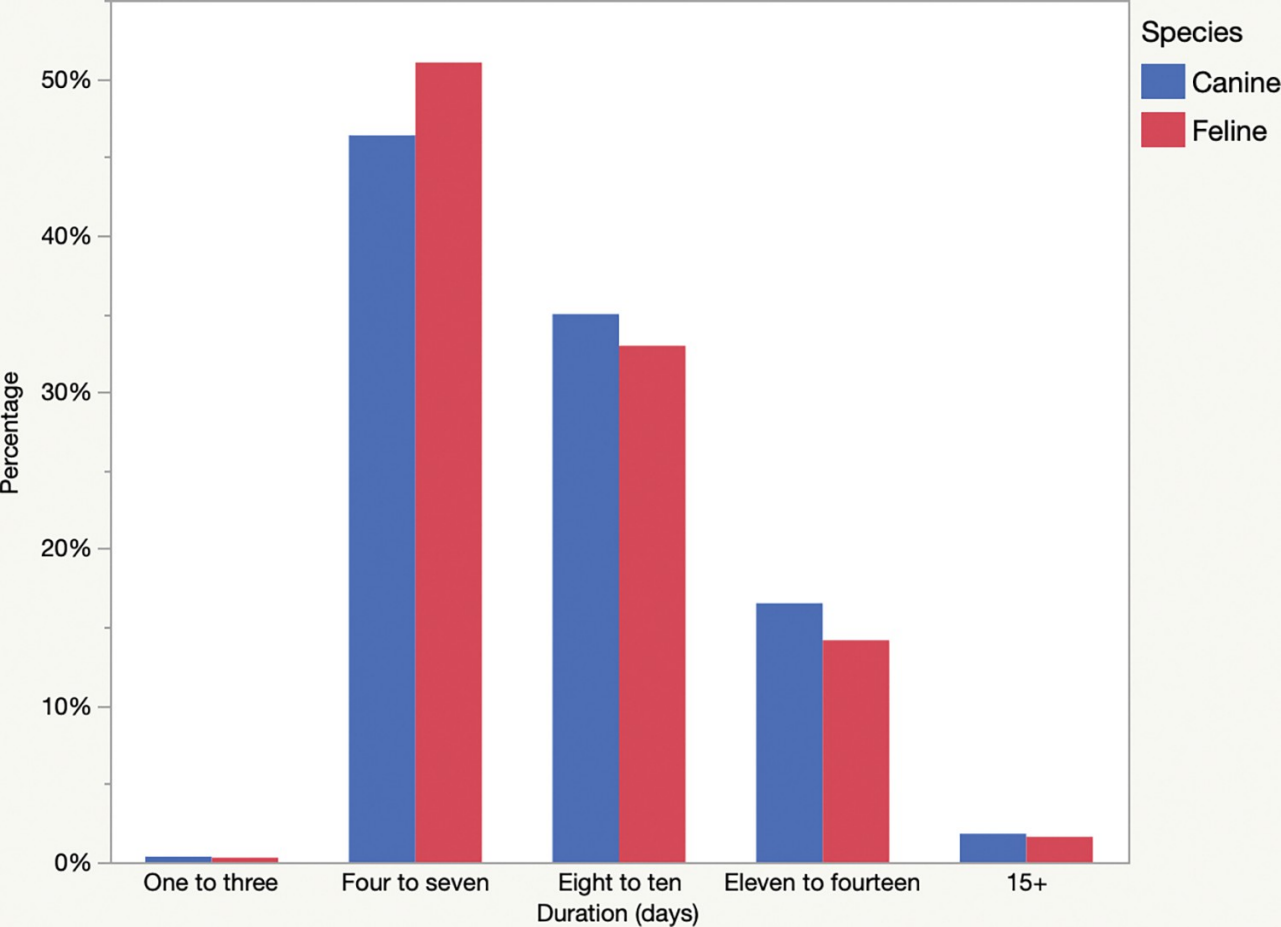
Duration of antimicrobial treatment (days) prescribed after dental procedures in dogs (n = 116,723; blue) and cats (n = 14,264; red). The x-axis denotes the duration of treatment in days, while the y-axis illustrates the percentage of cases.

Local or systemic antimicrobials were used in 116,723/713,901 (16.4%) procedures. The median duration of treatment with oral antimicrobials was ten days (IQR 3 days) (Fig 1). Durations for the most common antimicrobials are presented in Table 1. Duration (days) was positively associated with the diagnosis of periodontal disease of any stage (Stage 1 P = 0.04, Stages 2–4 *P*<0.0001). Conversely, extractions were associated with a shorter duration (median 7 vs 10 days, *P*<0.0001) of treatment.

**Table 1:** Median duration and interquartile range (days) for the antimicrobials most commonly administered to dogs (n = 116,723) and cats (n = 14,264) undergoing dental procedures.

Antimicrobial (n)	Dogs: Median (interquartile range)	Cats: Median (interquartile range)
Amoxicillin (4,966)	7 (5)	7 (3)
Amoxicillin clavulanate (19,175)	10 (7)	7 (3)
Cefpodoxime (17,017)	10 (7)	10 (3)
Clindamycin (45,128)	7 (3)	8 (3)
Metronidazole (3,329)	6 (2)	7 (5)
Doxycycline (973)	14 (19)	11.5 (4)
Marbofloxacin (916)	10 (4)	14 (4)

Using univariable analysis, age, weight, extraction of one or more teeth and diagnosis of periodontal disease (any stage) were associated with increased likelihood of antimicrobial administration (all *P*<0.0001). All of those variables were also significant in the multivariable model (Table 2).

**Table 2:** Multivariable model results for factors associated with antimicrobial use in dental procedures in dogs (n = 713,901).

Variable	Odds ratio	95% confidence interval	*P* value
Weight (kg)	1.004	1.003–1.005	<0.0001
Age (yr)	1.100	1.097–1.103	<0.0001
Periodontal disease stage (PD0-4)	Any stage vs none (PD0): 133.7	96.4–183	<0.0001
Extractions (number of teeth)	5.972	5.872–6.074	<0.0001

Antimicrobials were administered to 8.9% (52,905/591,496) of procedures in dogs that had no reported periodontal disease and no extractions, 38% (35,428/94,006) of procedures in dogs with no periodontal disease but one or more extractions, almost 100% (14,155/14,162) of procedures in dogs with any degree of periodontal disease and no extractions, and almost 100% (14,235/14,237) of procedures in dogs with extractions and any degree of reported periodontal disease.

1,057 clinics performed 100 or more procedures in the time of data gathering. Antimicrobial use in those clinics ranged from 1.5–98.8% of procedures (median 16%, IQR 11%). There was a trend towards decreased antimicrobial use by clinics that performed more procedures; however, this was not statistically significant (*P* = 0.087*)*.

#### Antimicrobial selection

The most commonly used antimicrobial and combinations are presented in Table 3. Of the canine patients that received antimicrobials, 2,623 (2.2%) received intravenous antimicrobials (cefazolin, ampicillin, ceftazidime, enrofloxacin, amikacin, gentamicin), alone or in combination with another antimicrobial. Four percent (4,669/116,723) of canine patients received doxycycline dental gel during the procedure. Over 99% (115,794/116,723) of treated dogs had antimicrobial therapy continued after discharge, either through an oral antimicrobial or having been administered cefovecin.

**Table 3:** Most commonly used antimicrobials or combinations of antimicrobials administered to dogs (n = 116,723) and cats (n = 14,264) during dental procedures.

Canine		Feline	
Antimicrobial drug or combination	n (%)	Antimicrobial drug or combination	n (%)
Clindamycin PO	48,045 (41%)	Cefovecin SC	7,098 (50%)
Amoxicillin clavulanate PO	20,081 (17%)	Clindamycin PO	3,387 (24%)
Cefpodoxime PO	17,508 (15%)	Amoxicillin clavulanate PO	2,559 (18%)
Cefovecin SC	11,249 (9.6%)	Amoxicillin PO	356 (2.5%)
Amoxicillin PO	5,214 (4.5%)	Doxycycline gel	110 (0.8%)
Metronidazole PO	3,497 (3.0%)	Marbofloxacin PO	97 (0.7%)
Doxycycline gel	3,111 (2.7%)	Metronidazole PO	91 (0.6%)
Doxycycline PO	995 (0.85%)	Orbifloxacin PO	55 (3.9%)
Clindamycin PO + doxycycline gel	976 (0.84%)	Cefazolin IV	55 (3.9%)
Marbofloxacin PO	957 (0.82%)	Cefovecin SC + doxycycline gel	44 (0.3%)
Cefazolin IV	625 (0.54%)	Doxycycline PO	40 (0.3%)
Cefazolin IV + clindamycin PO	592 (0.51%)	Cefazolin IV + clindamycin PO	37 (0.3%)
Ampicillin IV + clindamycin PO	512 (0.35%)	Cefpodoxime PO	34 (0.2%)
Ampicillin IV	302 (0.26%)	Cefovecin IV + clindamycin PO	32 (0.2%)
Cefazolin IV + cefpodoxime PO	262 (0.22%)	Ampicillin IV	31 (0.2%)

At the clinic level, cefovecin use ranged from 0–94% of procedures (median 3.0%), with 271 (25%) clinics not using the drug for any cases. There was no association between the number of procedures a clinic performed and use of cefovecin (*P* = 0.28).

Four hundred two (0.3%) canine patients received both cefovecin and oral antimicrobials. Seven (0.6%) clinics accounted for 23% of this use.

Decreasing weight and periodontal disease, but not dental extractions or age, were associated with post-procedure antimicrobials (oral antimicrobial or cefovecin) (data not presented).

Drugs classified as highest priority critically important antimicrobial (HPCIA) were administered to 26.5% (30,960/116,723) of canine patients.

Multivariable analysis of factors associated with the use of the main individual antimicrobials is presented in Table 4.

**Table 4:** Multivariable model results for factors associated with antimicrobial use in dental procedures in dogs that received antimicrobials (n = 116,723). Factors not associated with antimicrobial use are not presented in the table.

Antimicrobial	Variable	Odds ratio (95% CI)	*P* value
Amoxicillin	Age (yr)	0.98 (0.97–0.98)	<0.0001
	Weight (kg)	1.03 (1.03–1.04)	<0.0001
	Extractions (number of teeth)	0.69 (0.65–0.73)	<0.0001
	Periodontal disease stage (PD0-4)	Stage 1: 1.29 (1.14–1.45) Stage 3: 0.71 (0.62–0.81) Stage 4: 0.58 (0.47–0.71)	<0.0001 <0.0001 <0.0001
Amoxicillin clavulanate	Age (yr)	0.98 (0.98–0.99)	<0.0001
	Weight (kg)	0.98 (0.98–0.98)	<0.0001
	Extractions (number of teeth)	0.77 (0.74–0.79)	<0.0001
Cefovecin	Extractions (number of teeth)	1.16 (1.12–1.21)	<0.0001
Cefpodoxime	Age (yr)	0.99 (0.98–0.99)	<0.0001
	Weight (kg)	1.05 (1.05–1.05)	<0.0001
	Extractions (number of teeth)	0.16 (0.15–0.17)	<0.0001
	Periodontal disease (PD0-4)	Stage 2: 0.60 (0.55–0.65) Stage 3: 0.24 (0.22–0.28) Stage 4: 0.18 (0.14–0.22)	<0.0001 <0.0001 <0.0001
Clindamycin	Age (yr)	1.02 (1.02–1.03)	<0.0001
	Weight (kg)	0.97 (0.97–0.97)	<0.0001
	Extractions (number of teeth)	3.2 (3.09–3.26)	<0.0001
	Periodontal disease (PD0-4)	Stage 1: 1.10 (1.04–1.18) Stage 2: 1.49 (1.42–1.56) Stage 3: 1.76 (1.68–1.84)	<0.0001 <0.0001 <0.0001
Doxycycline dental gel	Age (yr)	1.03 (1.02–1.04)	<0.0001
	Weight (kg)	0.97 (0.97–0.97)	<0.0001
	Periodontal disease (PD0-4)	Stage 2: 1.35 (1.22–1.49) Stage 3: 1.35 (1.23–1.48)	<0.0001 <0.0001
Metronidazole	Weight (kg)	1.02 (1.01–1.02)	<0.0001
	Age (yr)	0.95 (0.94–0.96	<0.0001
	Extractions (number of teeth)	0.29 (0.27–0.32)	<0.0001
	Periodontal disease (PD0-4)	Stage 1: 0.82 (0.70–0.96) Stage 2: 0.74 (0.65–0.86) Stage 3: 0.52 (0.44–0.61) Stage 4: 0.31 (0.23–0.42)	0.015 <0.0001 <0.0001 <0.0001

#### Periodontal disease

Most canine patients (685,502, 96%) were not diagnosed with periodontal disease, while 4,663 (0.7%) had PD1 disease, 8,435 (1.2%) PD2, 10,059 (1.4%) SPD3 and 5,242 (0.7%) PD4. Antimicrobials were administered to 88,333 (13%) of dogs undergoing procedures with no periodontal disease, but 4,661 (99.9%), 8,431 (99.9%), 10,057 (99.9%) and 5,241 (99.9%) of dogs with periodontal disease stages 1 through 4, respectively (*P*<0.0001).

**Fig 2:**
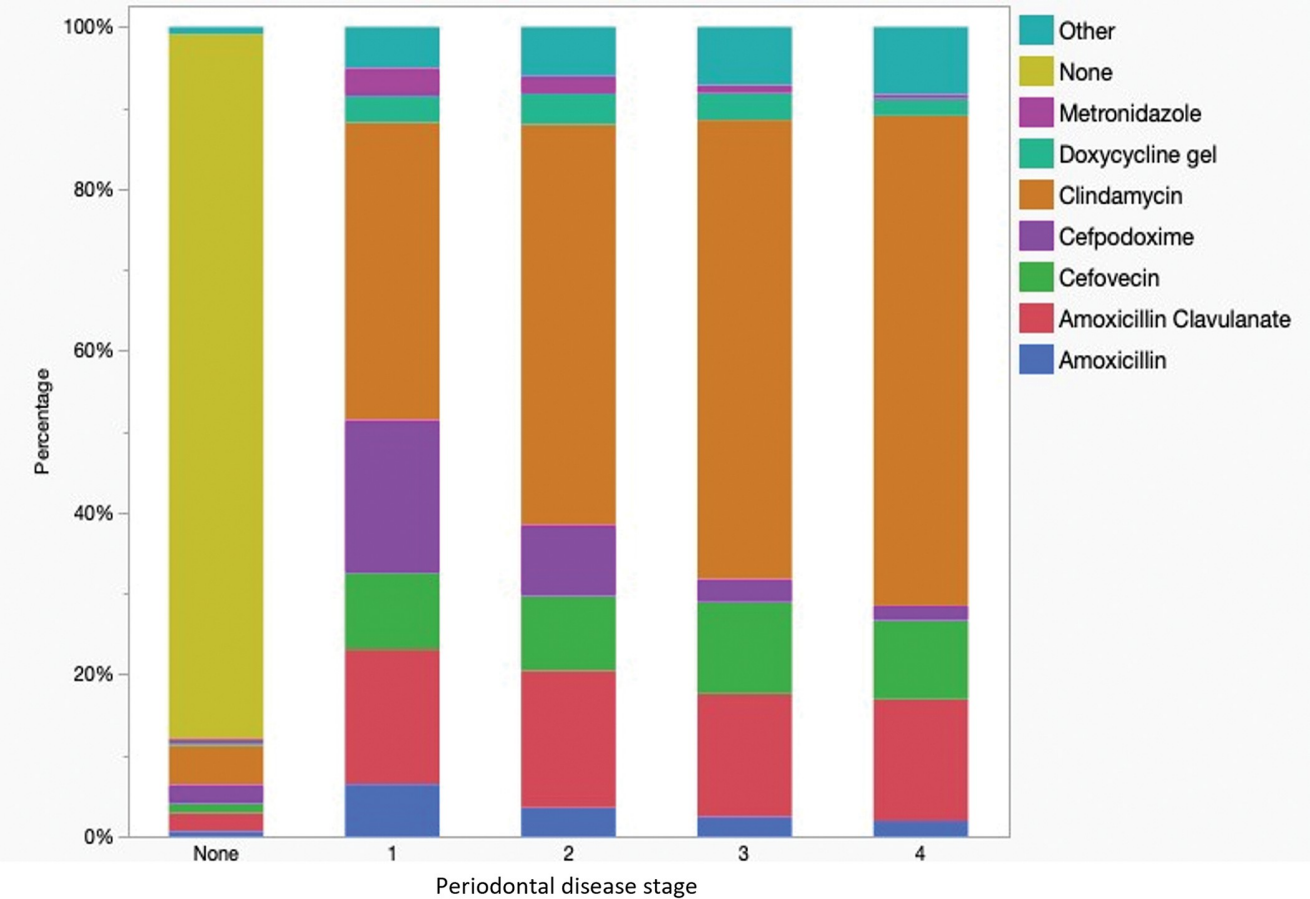
Antimicrobials used peri-procedurally in dogs undergoing dental procedures (n = 116,723), by periodontal disease stage (PD0-4).

There were significant differences in the main antimicrobial choices in dogs with periodontal disease (*P*<0.0001) (Fig 2, Table 2), with increases in the use of clindamycin (P<0.0001) and cefazolin (P<0.0001) and decreases in amoxicillin-clavulanate (P<0.0001), cefpodoxime (P<0.0001) and metronidazole (P<0.0001) in dogs with periodontal disease stages.

#### Dental extractions

**Fig 3:**
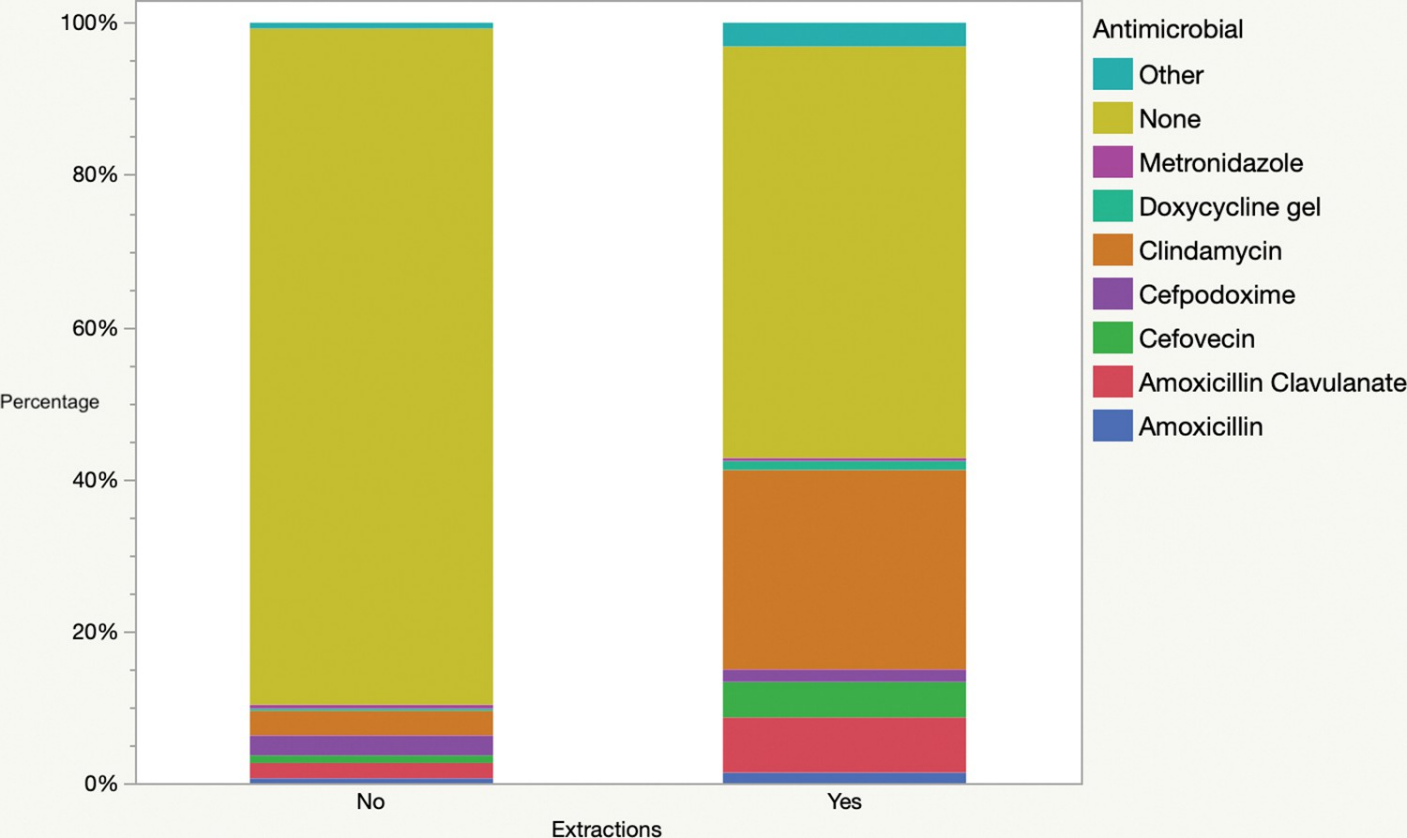
Comparison of the most common antimicrobials administered to dogs undergoing dental procedures that did, or did not, involve dental extractions.

**Fig 4:**
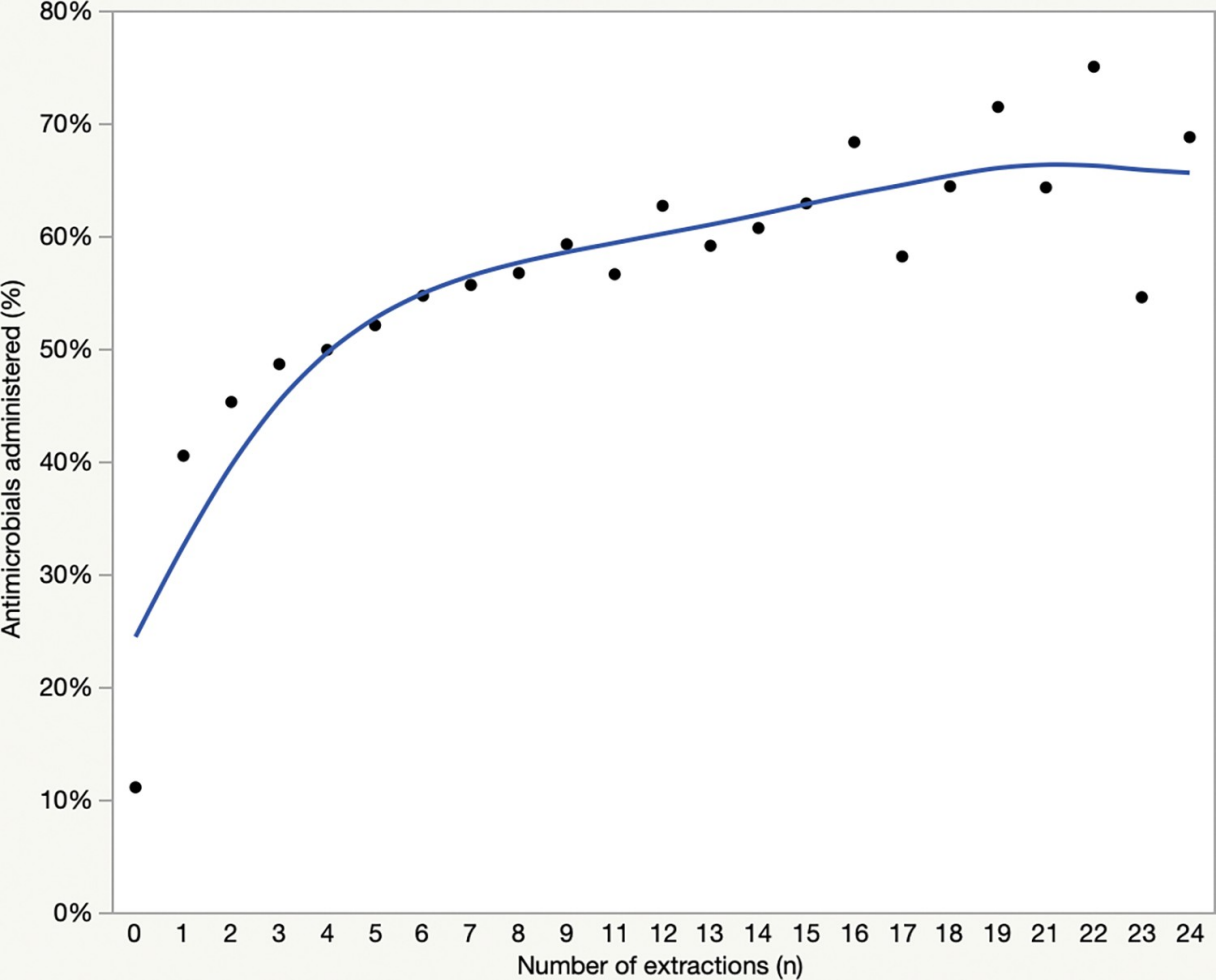
Relationship of the prevalence of antimicrobial administration to dogs undergoing dental procedures (n = 713,901) and the number of teeth that were extracted.

**Fig 5:**
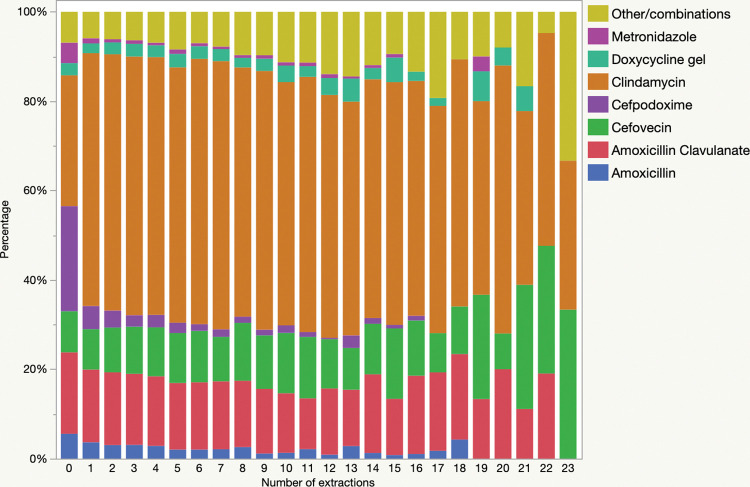
Comparison of peri-procedural antimicrobials that were administered to dogs undergoing dental procedures (n = 713,901), by the number of teeth that were extracted.

Extractions were performed in 108,243 (15%) of the procedures in dogs. The number of extractions ranged from 1–36 (median 2). Antimicrobials were administered to 49,663/108,243 (46%) of patients with extractions and 67,060/605,658 (11%) of patients that did not have extractions (*P*<0.0001). In procedures involving extractions, there were significant differences in antimicrobial use patterns (Fig 3, Table 2), with significantly more use of clindamycin and significantly less use of amoxicillin clavulanate, cefpodoxime and metronidazole (all *P*<0.0001). Corresponding to those changes, HPCIAs were significantly less commonly used in procedures involving dental extractions vs those that did not (14.8 vs 35.2%, *P*<0.0001). There was also a significant association between increasing number of teeth that were extracted and antimicrobial use (*P*<0.0001) (Fig 4). There was a significant association between number of extractions and antimicrobial selection (*P*<0.001), with significant decreases in single use of amoxicillin (P = 0.044), cefpodoxime (P<0.0001) and clindamycin (P = 0.0005) and a significant increase in antimicrobial combinations (P = 0.0004). Relative use of the main systemic antimicrobials is presented in Fig 5.

### Feline patients

Local or systemic antimicrobials were used in 14,264/104,249 (14%) procedures. Similar to dogs, age, weight, extraction of one or more teeth and diagnosis of periodontal disease (any stage) were associated with increased likelihood of antimicrobial administration using univariable analysis (all P<0.001). All of those variables were also significant in the multivariable model (Table 5).

**Table 5:** Multivariable model results for factors associated with antimicrobial use in dental procedures in cats (n = 104,249).

Variable	Odds ratio (95% confidence interval)	*P* value
Weight (kg)	0.95 (0.93–0.96)	<0.0001
Age (yr)	1.04 (1.03–1.05)	<0.0001
Periodontal disease (PD0-4)	6,558 (2,112–20,359)	<0.0001
Extractions (number of teeth)	6.8 (6.5–7.1)	<0.0001

**Fig 6:**
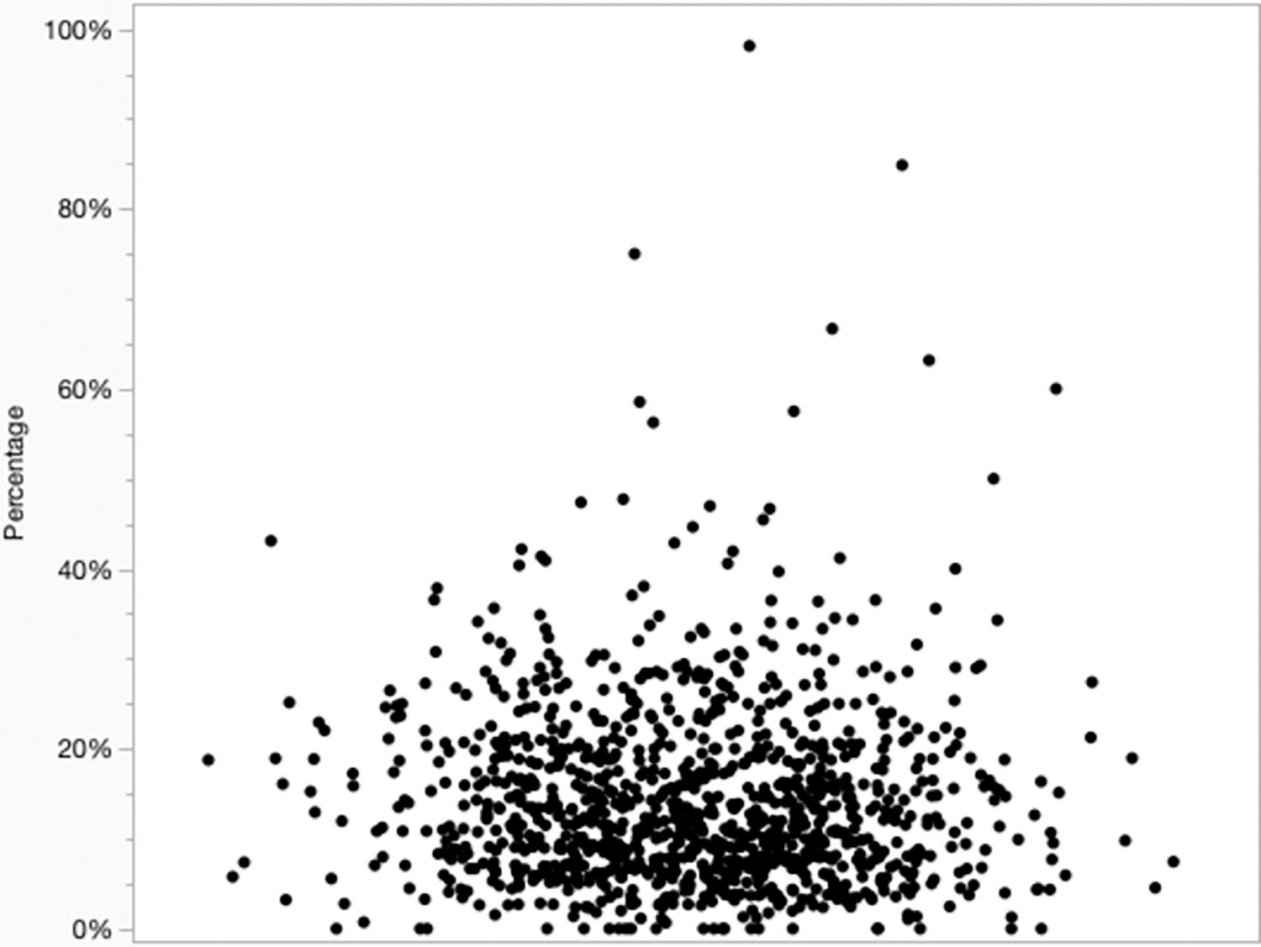
Scatterplot of the clinic-level prevalence of antimicrobial administration to cats undergoing dental procedures (n = 104,249). The clinics are spread out in the x-axis and the y-axis shows the percentage of cases that received antimicrobial therapy.

Four hundred fourteen (38%) clinics performed at least 100 procedures and antimicrobial use at the clinic level ranged from 1.0–98% (median 11%, IQR 11%) (Fig 6).

There was a significant impact of number of procedures performed by a clinic and antimicrobial use, with significantly greater use by clinics that performed fewer procedures (P = 0.0002).

#### Antimicrobial selection

Sixty-seven different antimicrobials and combinations were reported (including dental antimicrobial gels) but sole administration of cefovecin, oral clindamycin or amoxicillin clavulanate accounted for 13,044 (91%) of cases. The median duration of treatment with oral antimicrobials was seven days (IQR 3 days) (Fig 1). Durations for the most common antimicrobials are presented in Table 1. There was an association between periodontal disease and longer duration of treatment, but only for Stage 4 disease (*P* = 0.009). Duration of treatment was shorter for feline patients that underwent extractions compared to those that did not (7 vs 9 days, *P*<0.0001).

The most commonly used antimicrobials and combinations are presented in Table 3. Only 1.4% of treated cats (n = 209) received intravenous antimicrobials; one or more of cefazolin (n = 136), ampicillin (n = 62), clindamycin (n = 10), enrofloxacin (n = 9), ceftazidime (n = 2), amikacin (n = 1) and gentamicin (n = 1). Ninety cats (0.6%) received only an intravenous antimicrobial, while the others also received oral antimicrobials or cefovecin. One hundred eighty-eight cats (1.3%) were treated with doxycycline dental gel, alone or with other antimicrobials.

**Fig 7:**
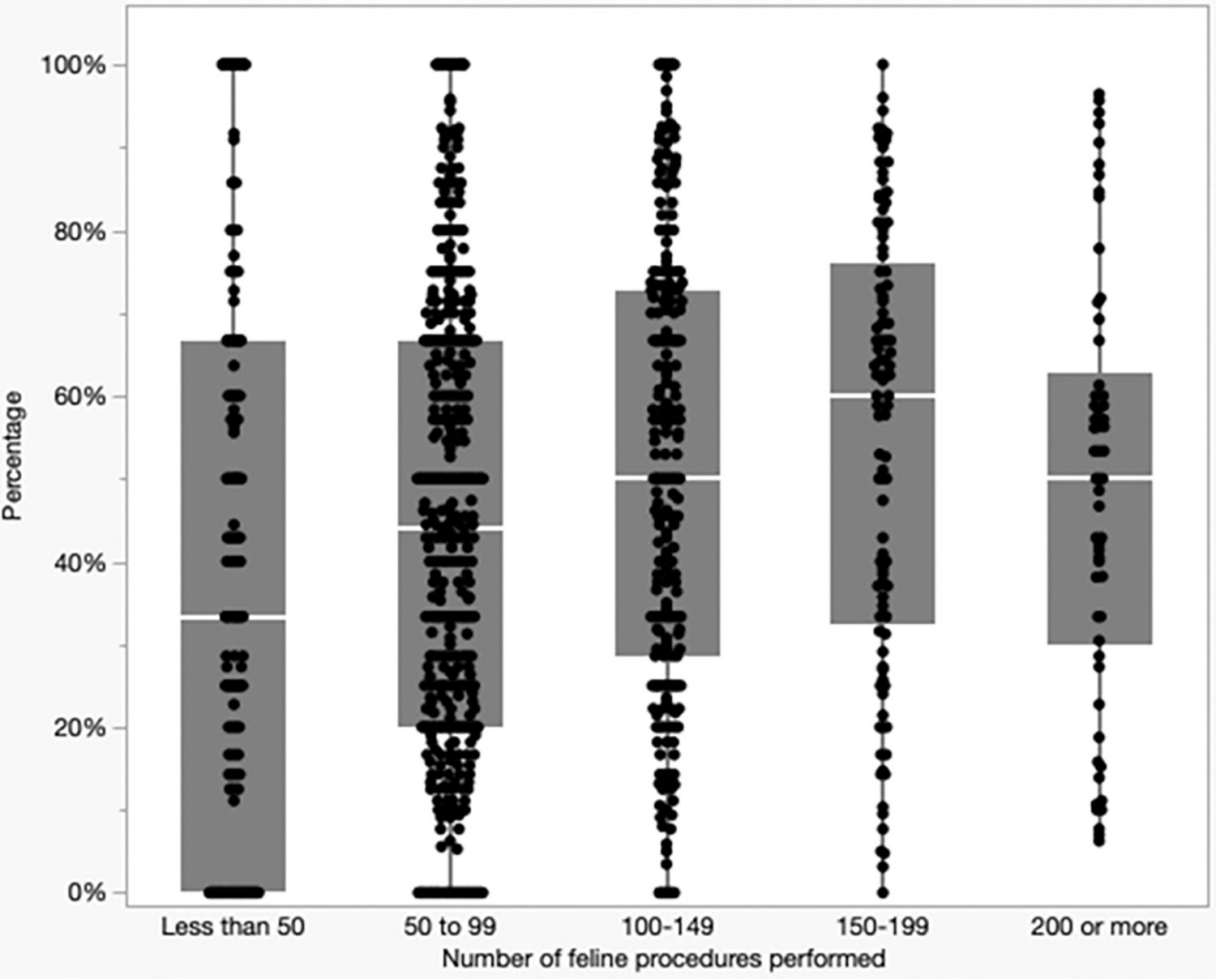
Clinic-level prevalence (%) of peri-procedural administration of cefovecin to cats undergoing dental procedures (n = 104,249).

Overall, 49% of treated cats (6,977/14,264) received one or more oral antimicrobials. Over 99% (14,174/14,264) of treated cats received an oral antimicrobial or cefovecin, or both. Fifty-nine (5.4%) clinics used cefovecin for all dental procedures, while 117 (11%) did not use it for any. In contrast to the association between the number of procedures a clinic performed and antimicrobial use, there was a positive association between increased procedure number and cefovecin use (*P*<0.001). The prevalence of cefovecin use based on categorization of the number of procedures performed at each clinic is presented in Fig 7. One hundred twenty-four (0.9%) of cats received both cefovecin and an oral antimicrobial.

Multivariable analysis of factors associated with the use of the main individual antimicrobials is presented in Table 6.

**Table 6:** Multivariable analysis of factors associated with use of selected antimicrobials in cats undergoing dental procedures (n = 14,264). Factors not associated with antimicrobial use are not presented in the table.

Antimicrobial	Variable	Odds ratio (95% CI)	*P* value
Amoxicillin	Extractions (number of teeth)	0.44 (0.34–0.56)	<0.0001
	Periodontal disease (PD0-4)	Stage 2: 0.40 (0.20–0.82)	0.012
Amoxicillin clavulanate	Age (yr)	0.98 (0.97–0.99)	0.0006
	Periodontal disease (PD0-4)	Stage 3: 0.75 (0.61–0.93)	0.007
	Extractions (number of teeth)	0.52 (0.47–0.57)	<0.0001
Cefovecin	Extractions (number of teeth)	0.85 (0.79–0.91)	<0.001
Cefpodoxime	Weight (kg)	1.41 (1.16–1.72)	0.0013
	Extractions (number of teeth)	0.31 (0.13–0.75)	0.0092
Clindamycin	Extractions (number of teeth)	2.5 (2.4–2.8)	<0.0001
	Periodontal disease (PD0-4)	Stage 2: 1.47 (1.26–1.71) Stage 3: 1.49 (1.28–1.74) Stage 4: 1.65 (1.31–2.08)	<0.0001 <0.0001 <0.0001
Doxycycline dental gel	None		
Metronidazole	Extractions (number of teeth)	0.34 (0.21–0.55)	<0.0001

Antimicrobials were administered to 7.8% (6,980/89,051) cats that had no reported periodontal disease and no extractions, 36% (4,471/12,382) of cats with extractions but no periodontal disease, 99.8% (1,404/1,407) cats with periodontal disease but no extractions and 100% (n = 1,409) cats with periodontal disease of any degree and extractions.

One or more antimicrobials classified as HPCIA were administered to 7,469 (52%) of treated cats.

#### Periodontal disease

Periodontal disease was diagnosed in 2.7% of cats; 740 (0.7%) of cats had PD1 disease, 885 (0.8%) PD2, 856 (0.8%) PD3 and 335 (0.3%) PD4. Antimicrobials were administered to 11,451/101,433 (11%) cats with no documented periodontal disease and 739/740 (99.9%), 884/885 (99.9%), 855/856 (99.9%) and 335/335 (100%) cats with stages 1 through 4, respectively (*P*<0.0001).

**Fig 8:**
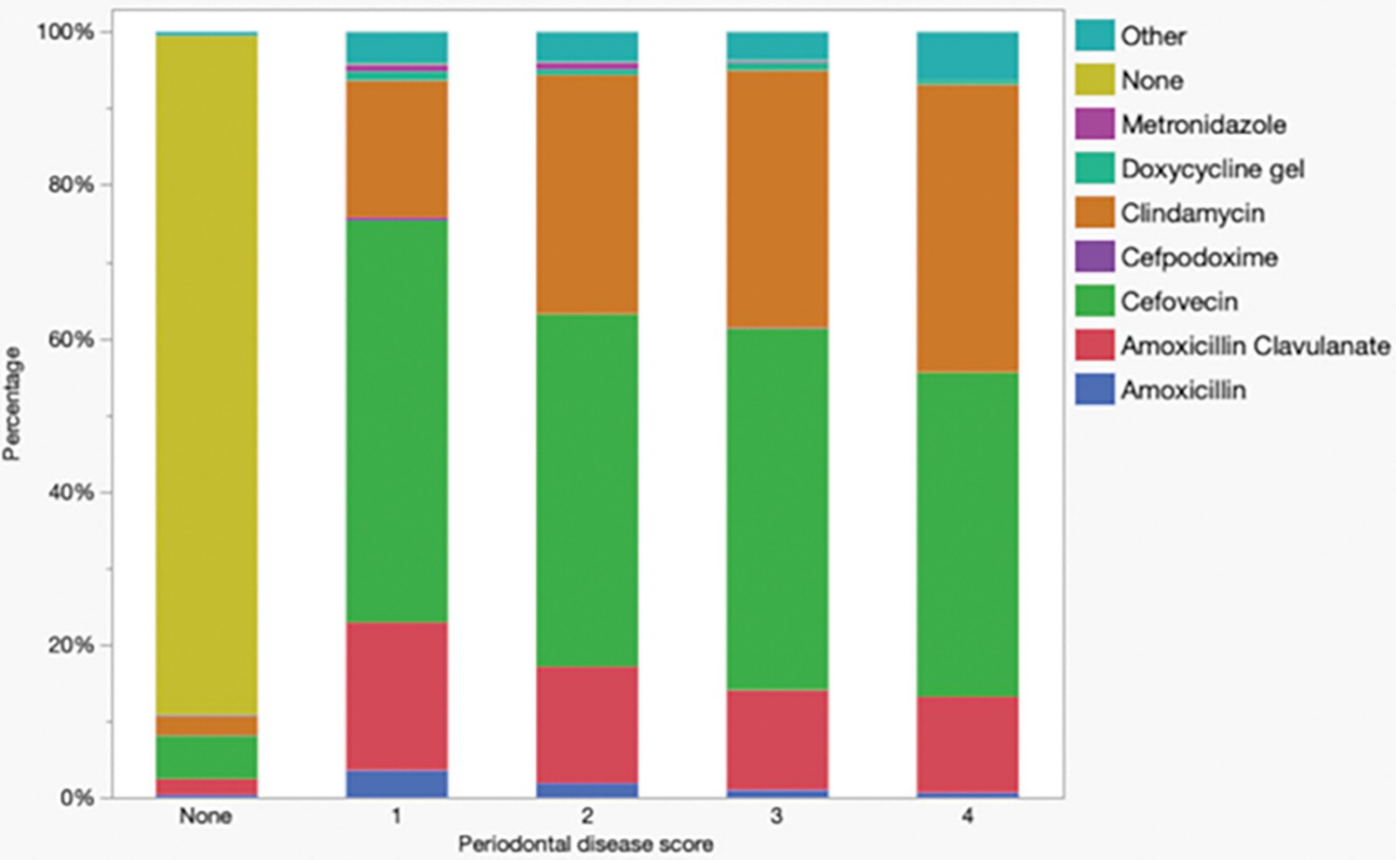
Antimicrobials used peri-procedurally in cats undergoing dental procedures (n = 104,249) by periodontal disease stage (PD0-4).

There were significant differences in the main antimicrobial choices in cats with periodontal disease (*P*<0.0001), with increases in the use of clindamycin (P<0.0001) and cefazolin (P = 0.01) and decreases in amoxicillin-clavulanate (P<0.0001), cefovecin (P = 0.013), and metronidazole (P = 0.47) in cats with increasing periodontal disease stages (Fig 8).

#### Extractions

Extraction of one or more teeth was performed in 13,791 (13%), ranging from 1–29 teeth (median 2, IQR 2). Antimicrobials were administered to 5,880/13,791 (43%) of cats with extractions and 8,384/90,458 (9.3%) of cats that did not have extractions (*P*<0.0001).

In cats that had extractions, there were significant differences in antimicrobial use patterns (*P*<0.001), with significantly more use of clindamycin, cefazolin and ampicillin, and significantly less use of cefovecin, amoxicillin, amoxicillin clavulanate, cefpodoxime and metronidazole (all *P*<0.01). Corresponding to those changes, HPCIAs were significantly less commonly used in cats undergoing dental extractions vs those that did not (49% vs 55%, *P*<0.0001). There was also a significant association between increasing number of teeth that were extracted and antimicrobial use (*P*<0.0001). There was a significant association between number of extractions and antimicrobial selection (*P*<0.001).

## Discussion

While it was encouraging that the majority of dogs and cats undergoing dental procedures did not receive antimicrobials, antimicrobial use was still relatively common, with frequent use of prolonged post-procedure regimens and use of higher tier drugs like 3^rd^ generation cephalosporins and fluoroquinolones.

There are currently no standard guidelines for antimicrobial prophylaxis in veterinary dentistry in the United States [12,13]. The American Veterinary Dental College indicates that patients may benefit from pre-treatment with antimicrobials ‘*to improve the health of infected oral tis*sues’ and that systemic antimicrobials are ‘*recommended to reduce bacteremia for animals that are immunocompromised*, *have underlying systemic disease (such as certain clinically-evident cardiac disease (subaortic stenosis) or severe hepatic or renal disease) and/or when severe oral infection is present*” [14]. American Animal Hospital Association guidelines indicate that antimicrobials ‘*may be indicated in patients with systemic risk factors*, *such as subaortic stenosis*, *systemic immunosuppression and orthopedic implants placed in the last 12–18 months*” [15]. These are more permissive than human guidelines, which recommend antimicrobials in a narrow patient population, focused mainly on prevention of infective endocarditis in patients with severe cardiac disease [16,17]. However, an approach similar to human dentistry has been recommended for veterinary patients [18], whereby antimicrobials are only indicated in animals with severe cardiac disease or abnormalities, namely patent ductus arteriosis, unrepaired cyanotic congenital heart defect, subaortic or aortic stenosis; the presence of embedded pacemaker leads; or, previous infective endocarditis [19].

Because of the lack of evidence and consistent guidelines, this study did not aim to assess compliance with standard practices. Rather, it aimed to describe the current state of antimicrobial use (AMU) in this population. Yet, even if the broadest approach is taken regarding indications for prophylactic AMU, antimicrobials may be used in excess of those recommendations. While the incidences of the above stated potential risk factors in the primary care dog and cat population is unknown, it is reasonable to assume that they are well under the prevalence of AMU noted here (16% and 14% in dogs and cats, respectively).

The striking inter-clinic differences in AMU also support the assumption that antimicrobials may be used unnecessarily, especially in the absence of clear professional guidelines. Some clinics virtually never used antimicrobials while some used them in almost all cases. While there may be differences in caseload and patient populations, these are unlikely to be profound and there is a relatively large subset of outliers that may account for overuse and are prime targets for interventions. Procedure complication rates were not available, but it is unlikely that antimicrobials are indicated in a large percentage of cases when many clinics rarely or never used them. Increased AMU for feline procedures was found in clinics that performed fewer dental procedures. While data about the specific veterinarians performing procedures was not available, this raises the question of whether clinicians at clinics that perform more procedures might have been more likely to obtain continuing education on the topic or be less prone to defensive prescribing because of greater confidence and experience as compared to veterinarians that perform dental procedures infrequently or less commonly.

This tendency to use antimicrobials is not particularly surprising given limited availability and visibility to antimicrobial guidelines for professional dental cleanings. Despite the presence of clear guidelines in human dentistry, auditing of dentists has shown gaps in understanding of guidelines [20] and low rates of appropriate AMU have been identified [21].

Periodontal disease was strongly associated with AMU. While periodontal disease stages were provided for all animals in this dataset, the study was not designed to report the prevalence of this condition, overall or by grade. All Banfield hospitals are equipped with digital dental radiography, but pet owners may decline specific services, such as full mouth radiography, which would confirm staging of periodontal disease. Underestimation of the true prevalence of periodontal disease in the absence of dental radiography has been reported in the veterinary literature [22,23]. Additionally, the absence of a diagnosis of periodontal disease could also mean that there was no structured code consistent with periodontal disease indicated at the time of the dental prophylaxis as opposed to the patient truly having no evidence of attachment loss. Therefore, these data do not necessarily accurately reflect the prevalence of periodontal disease in this population. However, this should not impact the evaluation of AMU practices since, regardless of whether or not the animal truly had periodontal disease, the focus was on antimicrobial decisions that occurred based on the veterinarian’s personal determination of whether periodontal disease was present and the severity of disease.

Dental extractions were also associated with AMU in both dogs and cats. While veterinary data are lacking, routine use of antimicrobials is not recommended in human dentistry when performing periodontal treatments or extractions, although use is fairly common [21].

Weight was associated with antimicrobial selections. However, the clinical relevance of this is probably limited given the small odds ratios that were present (e.g., 1.004 for antimicrobial use in dogs). With large datasets, numerically small and biologically irrelevant differences are more likely to be encountered. In contrast, the association of increased antimicrobial use with increased age was stronger and it is reasonable to assume that there was intended increased use of antimicrobials in older animals. Whether this was because of perceived risk, the more common presence of comorbidities or other factors was not investigated.

In addition to whether antimicrobials are used, evaluation of antimicrobial regimens is important for antimicrobial stewardship. Appropriate prophylaxis for dental procedures would involve use of pre-procedure intravenous antimicrobials [6] (which was uncommon) with rare need for post-procedure administration [24] (which was very common), particularly prolonged oral treatment or use of an injectable 3^rd^ generation cephalosporin with a long half life, which was given to 50% of treated cats and 9.6% of treated dogs. A basic principle of antimicrobial prophylaxis is that antimicrobials should be present at therapeutic levels during the period of risk, which corresponds mainly to the time of the procedure, when tissues are manipulated and bacteremia risks are higher, and potentially up to 24 hours after the procedures. The timing of administration of intravenous antimicrobials was difficult to determine precisely (pre-operative or peri-operative administration), and timing of oral and subcutaneous administration was also difficult to predict. Further, when used, oral antimicrobials were dispensed for a median of 10 days in dogs and seven days in cats, durations that would well exceed recommended prophylaxis for dental or surgical procedures in humans in which antibiotic treatment for 24 hrs or less has been shown to sufficiently in preventing the occurrence of postoperative infections after ear, nose, throat, and oral and maxillofacial surgery in healthy patients [25–27].

It is possible that the presence of comorbidities influenced decisions in some cases and comorbidity information was not included in the analysis. However, based on the size of the dataset, the likely low incidence of relevant comorbidities and the focus on medians and common practices rather the measures that would be more impacted by outliers, it is assumed that these data reflect common use practices and were not substantially impacted by outliers. However, even when comorbidities are present, antimicrobials may not be indicated, such as can be seen from human guidance that indicates the presence of prosthetic joint implants is not an indication for prophylactic antimicrobials in humans [28].

Local antimicrobials have been recommended for stage 2 or 3 periodontal disease in dogs and cats [15]. However, this was only used in 5.7–5.8% of dogs diagnosed with that disease, and only 1.4–1.6% of cats. Interestingly, while the diagnosis of periodontal disease was associated with local antimicrobials in dogs, there was no such association in cats, raising questions as to reasons behind local antimicrobial selection in cats. The efficacy of local antimicrobials in conjunction with scaling and root planning is unclear in humans and dogs [29,30], so it is difficult to assess the relevance of the use (or lack of use) of antimicrobial gels in this patient population.

A variety of antimicrobials were used; however, a limited number of antimicrobials predominated. Clindamycin, amoxicillin-clavulanate and amoxicillin were the most common oral antimicrobials. These have been recommended as first line options for treatment of dental infections [18], given their effectiveness against main opportunists found in the oral cavity and their lower tier status [31]. However, while the drug selection is encouraging, the predominance of oral and subcutaneous antimicrobials does not align with the goals of prophylaxis. Cefovecin was widely used, particularly in cats. This is a higher tier drug that has a prolonged duration of activity. There are indications for this drug therapeutically, particularly for animals that need a broad-spectrum antimicrobial and cannot be medicated orally, or there are concerns regarding compliance or negative impacts to the human animal bond [32,33]. However, it is difficult to justify the use of this drug for routine dental prophylaxis, when only pre-operative use of a more narrow spectrum drug would be required in most routine cases. Clindamycin was preferentially used when extractions were performed, with decreased use of beta-lactams and metronidazole. Reasons for this are unclear but may relate to a preference for an antimicrobial with very good activity against anaerobes and in bone [34]. However, metronidazole has similar characteristics [35,36] and was used less in animals that underwent extractions or that had periodontal disease. Reasons for selection of different drugs provide an opportunity for future study.

These data provide comprehensive information about use of antimicrobials during dental procedures in primary care veterinary practices. It is possible that the initial search for cases was incomplete, as the search was based on completed procedural invoice items. It is highly unlikely that every dog or cat with periodontal disease had a dental prophylaxis in 2020. It is also important to note that this study focused on the decision making related to periodontal disease stage and the number of extractions being performed on these patients. Thus, further research looking at other factors for antimicrobial use and drug selection such as other dental and maxillofacial diseases, diagnostic imaging findings, and for patients with co-morbidities is warranted.

## Conclusions

In conclusion, this study offers valuable insights into the utilization of antimicrobials in dental procedures within primary care veterinary practices. While the research primarily examined decision-making processes concerning the diagnosis of periodontal disease, as well as the extent of extractions performed on patients, it sheds light on critical areas necessitating further investigation. These areas include understanding the rationale behind antimicrobial usage and drug selection for various dental and maxillofacial conditions, especially in patients with coexisting medical conditions. Additionally, the study underscores opportunities for intervention, such as reducing post-dental antimicrobial administration, which aligns with the principles of antimicrobial stewardship. Such interventions aim to promote prudent use of antimicrobials in veterinary dentistry.
